# Factors influencing sport persistence still represent a knowledge gap – the experience of a systematic review

**DOI:** 10.1186/s40359-024-02098-6

**Published:** 2024-10-23

**Authors:** Karolina Eszter Kovács, Zsolt Szakál

**Affiliations:** 1 University of Debrecen, Faculty of Arts, Institute of Psychology, Debrecen, Hungary; 2 Debrecen Reformed Theological University, Kölcsey Ferenc Teacher Training Institute, Debrecen, Hungary

**Keywords:** Sport persistence, Commitment, Sport participation, Ecological model, Systematic review

## Abstract

Investigating sport persistence can make a significant contribution to a thorough examination and understanding of the factors underlying an athlete’s activity, integrating physical activity, sports motivation and commitment. This systematic review aimed to detect the papers focusing on the factors influencing sport persistence following the levels of Bronfenbrenner’s and Bauman’s ecological models. Following the PRISMA guidelines, by the EBSCO Discovery Service Search Engine, 512 records were detected, of which 21 met the criteria. Factors influencing sport persistence show a huge variety. Individual factors and micro-system level were overrepresented. Meso- and macro-level factors are underrepresented and measured only with other (individual or micro-system level) factors. Most factors detected at the individual (e.g. gender, age, career-related variables and positive psychological factors), micro- (e.g. family, peers and coaches), meso- (e.g. climate), and macro-level (cultural, societal, and institutional influences) were positively correlated with sport persistence, i.e. supporting the persistent behaviour of the athletes regarding participating in one’s sporting activity. In conclusion, there is still room for further research concerning sport persistence. Including variables belonging to the meso- and macro-system and conducting longitudinal research should be considered which would allow us to better understand the nature of sport persistence.

**Supplementary Information:**

The online version contains supplementary material available at 10.1186/s40359-024-02098-6.

## Introduction

It is well-known that participation in sport has a positive impact on a person’s lifestyle, health and health-awareness, regardless of gender and age [1, 2]. Participation in sport from childhood contributes to psychophysical development and the acquisition of a healthy lifestyle maintained in later life [3, 4]. Regular and optimally pursued (i.e. not as an addictive behaviour) sporting activities can develop a range of psychological skills, including motivation, activity, self-discipline, perseverance, or realistic self-evaluation [5, 6].

To understand the nature of sport persistence, it is worth starting with the most basic definitions. In the last decades, the definition of sport had a significant change. The diversity of definitions surrounding the concept of sport stems from its multifaceted nature, influenced by cultural, historical, and societal factors, as well as varying perspectives on the purpose, structure, and significance of physical activities. The European Charter of Sport (1997) defines sport as “any physical activity, whether performed occasionally or in an organized form, the purpose of which is to develop or improve physical and mental fitness, to create social contact or to achieve results in competition at various levels.” [7]. Exercise is a narrower term defined as “a specific type of physical activity that is planned, structured and repeatedly done to improve or maintain physical fitness, whereas the definition of physical activity covers “any movement produced by skeletal muscles that result in energy expenditure” [8, p. 128]. Physical activity, defined by the WHO [9], is any bodily movement produced by skeletal muscles that requires energy expenditure. Physical activity refers to all movement carried out during leisure time, for transport to and from places, or as part of a person’s work. Both moderate- and vigorous-intensity physical activity improve health. Since it is crucial to start physical activity at an early age, physical activity has an essential role too. “Physical education is a purposeful, planned educational process which, with its specific educational content (movement games, physical exercises, sports action systems and the corresponding theoretical knowledge) is aimed at developing a well-rounded personality, based on proactively defined social goals” [10, p. 37]. It has educational content, formal and substantive features, and a system of tools.

An indicator of performance may be sport persistence, a complex concept, the terminology used to express the mastery, form, level and effectiveness of the sporting activity. However, its use is less common as theories tend to focus on sport motivation and commitment, which do not fully correspond to the term sport persistence since it combines the physical, mental and social aspects of the sport together [11]. It goes beyond sport motivation and commitment; it is the embodiment of sport performance and mental toughness. Sport persistence can be coded as the peak of sport performance and commitment. It incorporates psychological skills such as resilience [11], adaptive and proactive coping [12, 13] and positive personality characteristics that can also contribute to preventing drop-out, and enabling athletes to show greater training commitment [14]. Thus, the athlete is not merely committed to the sporting activity but is qualitatively committed to it. It refers to the attempts we make to resolve, process and use stressful situations associated with the performance plateau, failures, injuries or even successes and positive events. This behaviour and performance are covered by the term sport persistence, which is not widely studied in international practice, as research typically focuses on sporting habits, sport motivation and commitment [12, 13]. Overall, following the definition of sport persistence, it can be considered a performance indicator while it refers to the achievement of the person through the sustained physical activity (regardless the level of activity).

The role of persistence is unquestionable in competitive sport, as a successful sporting career can only be achieved if the athletes persist in their activity, professionally, with individual and coach-led training, ideally combined with sport psychological support. From a sport and personality psychological context, the reinforcement of grit and persistence is of particular relevance, as available research suggests that grit is positively correlated with competitive performance and time spent on training [15, 16], deliberate practice and self-improvement [17], success in decision-making tasks [15], and athletes’ commitment to sport [17]. Accordingly, it is inversely related to the extent of thoughts about dropping out of sport or changing the type of sport [17]. In competitive sport, dropout prevention is of particular importance, as it takes time to reap the rewards of persistent effort. However, the significant physical, emotional and mental changes during adolescence can significantly complicate the process, often causing a (temporary) lack of results. National experience shows that dropout from sport often occurs before peak performance is reached [18]. However, sport persistence is worth being investigated in the case of recreational sports as well. Recreational sport often lacks a competitive component and is not as effective as competitive sports in terms of performance since there are no tangible indicators of achievement such as medals or recognition. At the same time, the role of sustained and persistent recreational sport is of particular importance for personality development, especially in sensitive periods such as adolescence or young adulthood, as the effects of persistent sport participation, although with a different focus, can also be detected in recreational sport [18].Fundamentally, sustained sporting activity can be influenced by several factors determining the mode, frequency and type of sporting activity pursued. Individual, micro-, meso- and macro-system-related factors all have a long-lasting and significant influence on the characteristics of the physical activity a person engages in. Cairney et al. [14] emphasise the role of social participation, enjoyment of the activity and physical competence, in addition to an individual motivation to participate in sport, which is mutually dependent. Individual, micro-, meso- and macro-system-related factors result in physical, mental and social health, following the components of the bio-psycho-social model of health [19]. The model specifically highlights the role of habitus, physical endowments, professionalism and academic achievement, which are influenced by the form, level and engagement of the sporting activity undertaken, and which, through the cycle, are also affected by these attributes either positively or negatively.

Beyond regular sporting activity, but based on it, commitment to sport represents a higher level. In this case, the individuals, building on their characteristics and strengths, actively participate in sporting activities to overcome disadvantages [20]. As with regular participation in sport, motivation is the cornerstone of sport engagement and determines the extent and quality of engagement [21, 22]. Scanlan et al. [23] draw on the aforementioned determinants of sport participation in their sport commitment model. Among the individual factors, sport enjoyment appears like a basic segment, but the role of personal investments and valuable opportunities is also highlighted. However, opportunities given to the individual and other priorities may distract the athlete from sporting activity, even leading to dropout in the long term. Combining these factors will determine the athlete’s choice to commit more strongly to a sport or a civic profession and/or education. In social terms, the role of peer support in persistent sporting behaviour is a well-known fact, whether it is the support of family, the peers or the sports club. However, the effect of peer pressure, which refers to the expectations and norms of society, should also be mentioned as a potential factor supporting sport persistence [23, 24].

Sport commitment is the basis for the main concept of the research, known as sport persistence [20]. Sport persistence can ultimately be defined as a product of sport performance and commitment. It implies personality traits integrating resilience, adaptive coping and positive personality, i.e. the athlete is not simply persistent in sport but qualitatively committed to it. The term covers this behaviour and performance [25]. Still, the study of sport persistence is not widespread in international practice, as research typically focuses on sporting habits, sport motivation and commitment. In addition, a shortcoming of the existing research is that sport persistence is considered a bivariate variable. Thus, athletes are included in the persistent group if they are still playing sport at the time of the study, i.e. has not dropped out. However, it would be important to capture persistence more precisely by creating a complex indicator [26]. In our previous research, we attempted to do this on a sample of higher education students, integrating into the indicator elements such as whether the students had won a sports scholarship, whether they had received extra credit for sports performance during the higher educational admission, whether they were a member of a sports club/sports association, and also the current sports participation frequency [13].

Based on the research results on sport participation, both nationally and internationally, and across sporting levels, it is worth addressing the issue of persistence. Investigating the sporting habits, sport commitment and persistence of athletes learning in any kind of educational level is also essential to understand the balance between the area and roles of the athlete [27]. For this reason, especially in the case of competitive athletes, dealing with the dual career model of student-athletes is crucial for several reasons. Firstly, it recognizes the unique challenges faced by individuals striving to excel both academically and athletically simultaneously, shedding light on the intricate balance required between sporting behaviour and educational pursuits [28]. Understanding this model aids in developing tailored support systems, ensuring student-athletes can thrive in both domains without sacrificing one for the other. Moreover, exploring this concept fosters insights into optimizing training schedules, academic accommodations, and career transition strategies, ultimately enhancing the holistic development and well-being of student-athletes as they navigate their dual careers and beyond.

The model of Bauman et al. (2012) uses a multi-level comprehensive framework that classifies each of the variables that influence physical activity into levels. Due to the model’s ecological nature, it includes individuals’ interactions with society and their physical environment. The key principle is that a comprehensive understanding of the determinants of physical activity provides the opportunity to develop multi-level action plans with a significantly higher likelihood of success. The model incorporates individual variables such as biological and psychological factors and allows one to study the variables related to the micro-, meso-, exo-, and macro-system in a broad range of contexts. The combination and interaction of political, environmental, and global factors are considered to have a wide range of influences on sporting activity, but these have only been tangentially studied.

The purpose of the present systematic review is to review the results of previous studies concerning sport persistence. Therefore, the aim of this research is to investigate the factors that influence sport persistence among university students based on Bronfenbrenner’s and Bauman’s ecological model [29], which will allow for a more complex understanding of the concept of sport persistence. Based on this aim, we formulated the following research question: What factors can be detected to significantly influence the sport persistence (i.e. persistent participation in any sporting activity) of athletes younger than 25 years regarding the individual, micro-, meso-, and macro systems?

## Methods

This systematic literature review follows the Preferred Reporting Items for Systematic Reviews and Meta-Analyses (PRISMA) guidelines [30].

### Literature search

Systematically searches were conducted in EBSCO Discovery Service Search Engine, which contains 85 databases. The keywords we used for searching were “sports persistence”, “sport persistence”, “sport resilience”, “sports commitment”, “commitment to sport” AND “influential factors”, “individual factors”, “intrapersonal factors”, “Interpersonal factors”, “environmental factors”, “institutional climate”. The searches were performed on 16 December 2022. Our systematic searches resulted in a total of 512 records; after double filtering, 51 records were excluded. After abstract and title screening, 389 records were excluded. Therefore, 72 papers were sent for full-text screening which led to involving 21 papers in the qualitative synthesis.

### 2.2 Inclusion and exclusion criteria

In the current review, we focused on the factors influencing sport persistence. In our definition, sport persistence is the unwavering commitment and determination displayed by athletes to overcome challenges, setbacks, and obstacles in pursuit of their athletic goals. It encompasses resilience, mental toughness, and the ability to consistently push boundaries, regardless of difficulties encountered, to achieve success in sports (Kovács, 2021).

The following inclusion criteria were set:

1. original empirical research published in a peer-reviewed journal;
2. age below 25 years (due to focusing on students learning in primary, secondary or tertiary education);
3. participants pursuing regular physical activity or any kind of sport (competitive or leisure activity).
4. examined (the elements of) sports persistence;
5. examines at least factor influencing sport persistence (onto-, micro-, meso- or macro level);
6. written in English language, and.
7. in disciplines of education, psychology, health and medicine, social sciences and humanities and sports sciences.

Studies were excluded if they were.

1. Reviews, commentaries, letters to the editor, conference papers, books, book chapters, dissertations and newspaper articles
2. Focusing on non-healthy participants

### Data extraction and assessment of methodological quality

The authors independently searched the literature and reviewed study titles and abstracts. Then, the authors screened the titles and abstracts of all identified records. Studies that met the inclusion criteria were assessed for full-text review. The authors were responsible for the detailed analysis, quality assessment and data extraction of the included studies. In cases of uncertainty, both authors discussed the decision.

### Risk of bias

The quality of the studies was evaluated by the Joanna Briggs Institute (JBI) critical appraisal tool [31]. This tool assesses various aspects of study design, conduct, and reporting to gauge the reliability and validity of findings. It considers factors such as randomization, blinding, sample selection, and data analysis methods (see Appendix, Table A1). By identifying potential sources of bias, researchers can better interpret study outcomes and make informed decisions about the applicability of evidence in healthcare practice. This measure aids in promoting transparency, rigor, and credibility in research, thereby enhancing the quality of evidence-based healthcare interventions and guidelines. Papers were evaluated according to the appropriate tool on a 4-point scale (yes/no/unclear/not applicable) (Appendix, Table A1).

## Results

**Fig. 1:**
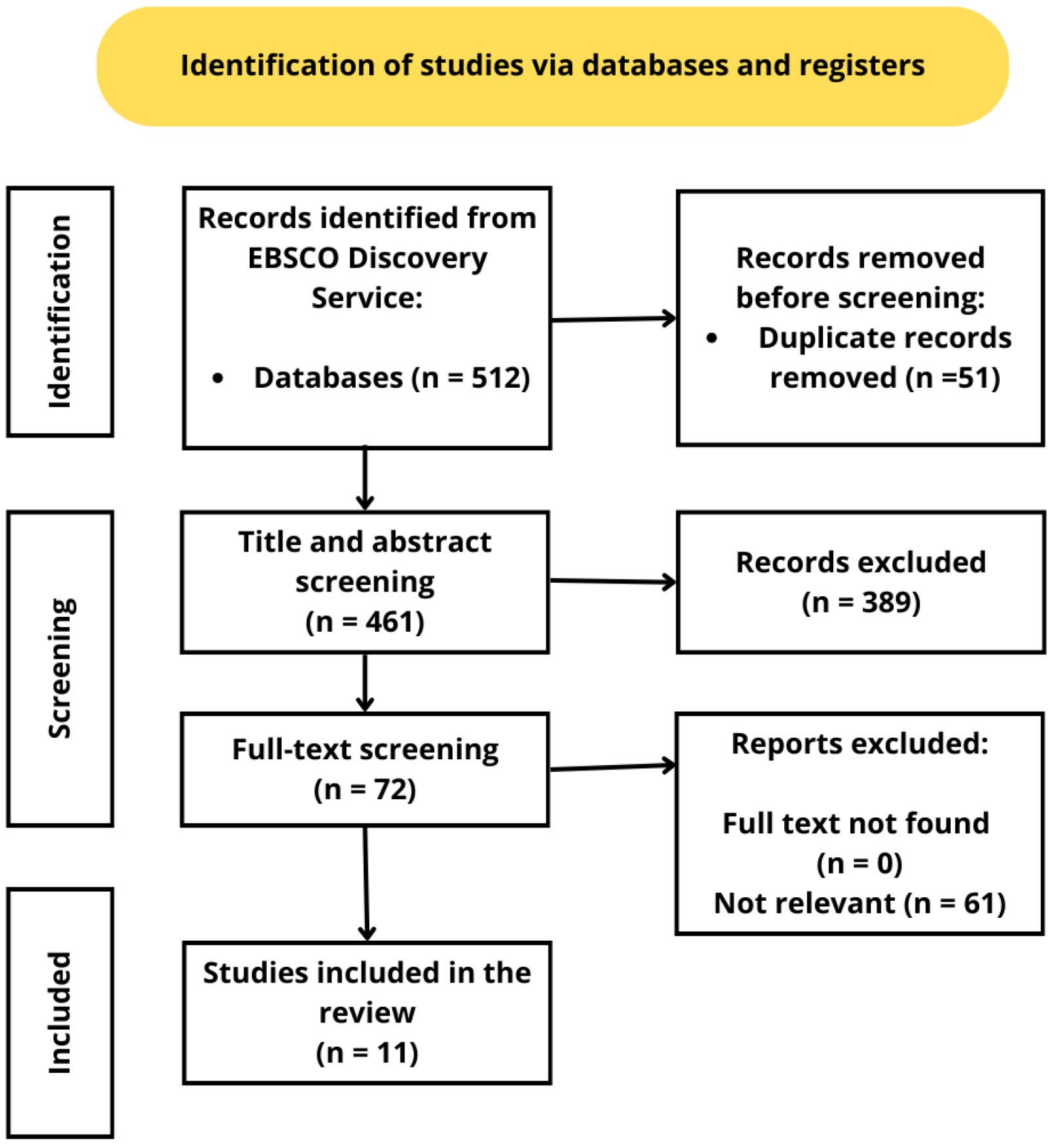
Preferred reporting items for systematic reviews and meta-analyses (PRISMA) diagram

Figure 1 introduces the research flow on the PRISMA diagram. Overall, 21 articles met the criteria (Table 1). The articles were published between 1997 and 2022, but approximately 2/3 of the papers were published after 2010 (*N* = 14). Most studies focus on one particular nation or country and only one paper focuses on two countries [32]. Spain was the most popular country with four investigations were carried out in this country [32–35]. Three studies were carried out concerning the United States [36–38], France [39–41] and Australia [42–44]. Two studies were introduced in regard of Canada [45, 46] and Germany [47, 48] while one-one study was conducted in Estonia [49], South-Korea [50], Slovenia [51], Italy [32] and Ukraine [52].

**Table 1:** Papers involved in the systematic review

		Country	Age group	Age	Level of education	Level of sport	Type of sport	Methodology	*N* of participants
Ahn et al.	2016	South-Korea	young adults	M = 23.5 (SD = 0.8)	tertiary	not elite athletes	general	quantitative	418
1. Albert et al.	2019	USA	adolescents	M = 15.81 (SD = 0.82)	secondary	not elite athletes	football	quantitative	81
2. Baron-Thiene & Alferman	2015	Germany	adolescents	M = 16.2 (SD = 0.65)	secondary	elite athletes	mixed (track and field, swimming, and diving in the summer and cross-country skiing, biathlon, ice skating, basketball, handball, soccer, volleyball)	quantitative	125
3. Calvo & Topa	2019	Spain	adolescents, young adults	M = 15.59 (SD = 2.38)	mixed	not elite athletes	football	quantitative	151
4. Cervelló et al.	2007	Spain	adolescents	M = 17.9 (SD = 1.3) M = 16.7 (SD = 1.1)	unknown	elite athletes	general, mostly tennis	quantitative	134
5. Consoni et al.	2021	Italy, Spain	adolescents	N/A (14–18 years)	secondary	not elite athletes	general	quantitative	614
6. Eime et al.	2014	Australia	adolescents	M ± SD = 12.2 ± 0.5 M ± SD = 16.2 ± 0.6	secondary	not elite athletes	general	quantitative	440
7. Fraser-Thomas et al.	2008	Canada	adolescents	M = 15.59 (SD = 2.38)	unknown	elite athletes	swimming	qualitative	20
8. Gucciardi & Jackson	2015	Australia	young adults	M = 16.4 (SD = 2.6) M = 18.3 (SD = 4.1)	unknown	not elite athletes	general	quantitative	292
9. Guillet et al.	2002	French	adolescents	M = 18.03 (SD = 1.29)	unknown	elite athletes	handball	quantitative	723
10. Joessar et al.	2011	Estonia	adolescents	M = 17.06 (SD = 1.32) M = 15 (SD = 0.81)	unknown	elite athletes	team sports	quantitative	424
11. Lea & Branco	2020	Slovenia	adolescents	M = 13.19 (SD = 1.56)	unknown	elite athletes	athletics (javelin, high jumping, running, sprint)	quantitative	566
12. Le Bars et al.	2009	French	adolescents	M = 14.87 (SD = N/A)	unknown	elite athletes	judo	quantitative	186
13. Lukwu & Luján	2011	Spain	adolescents	M = 15.6 (SD = 1.35)	secondary	elite athletes	handball	quantitative	302
14. Miller & Siegel	2017	USA	young adults	M = 35.35 (SD = 10.66)	unknown	not elite athletes	general	quantitative	234
15. Pelletier et al.	2002	Canada	adolescents	M = 15.6 (SD = N/A) from 13 to 22 years	unknown	elite athletes	swimming	quantitative	369
16. Popovich et al.	2022	Ukraine	young adults	N/A	unknown	not elite athletes	mixed (athletics, boxing, and weightlifting, mini-football, football, handball, and volleyball)	quantitative	634
17. Ryan et al.	1997	USA	young adults	M = 21 (SD = N/A)	tertiary	not elite athletes	aerobic, Tae Kwon Do	quantitative	40
18. Sarrazin et al.	2002	French	adolescents	M = 14.07(SD = 0.79)	unknown	elite athletes	handball	quantitative	335
19. Vella et al.	2014	Australia	adolescents	M = 8.25 (SD = 0.44)	primary	not elite athletes	general	quantitative	4042
20. Westmattelmann et al.	2021	Germany	young adults	M = 28.59 (SD = 22.02)	tertiary	both elite and non-elite athletes	general	quantitative	753

Quantitative studies were significantly more popular concerning the type of the investigation since 36 out of 21 papers introduce the results of quantitative studies and only one paper reflects on the results of interviews as a qualitative study method.

### Individual variables in the background of sport persistence

#### Non-psychological variables

Firstly, we investigated the non-psychological variables (including sociodemographical and sport-related individual factors) mentioned in the papers. We started the exploration of the sociodemographic variables. Most studies involved both male and female athletes in the investigations, however, some study focused only on women [39, 40, 43], on male athletes [38] or the gender distribution of the sample is unknown [36, 50, 51]. Even if most study contained information on the gender distribution of the sample, only a few introduced measurements on the impact of gender. Most found that drop-out, and persistence therefore is independent of gender [32, 37, 41, 45, 49]. Baron-Thiene & Alferman [47] stated that dropout is significantly higher among female athletes and Vella et al. [42] also detected that sports participants were more likely to be boys.

Concerning *age*, we could see that 14 papers focused on children and adolescents [32–34, 38–43, 45–47, 49, 51], six papers involved young adults or adults [36, 37, 44, 48, 50, 53] and one paper investigated both children and adults [35]. Table 2 shows the level of education they participate. Most studies do not consider level of education important in sport persistence since they do not have any information on the current stage of education. This can be because these papers rather focus on sports club membership instead of participation in education. which can also be an important interpreting variable, 12 studies investigated athletes who were members of a sports club or association [33, 35, 38–41, 44–47, 49, 51], three papers, besides members of a sports club or association, also involved athletes who were not members of a sports club or association [34, 48, 53] and five papers did not involve sport club members in their investigations or did not provided sufficient information regarding this variable [32, 37, 42, 43, 50].

**Table 2:** The factors influencing sport persistence in the various levels of the ecological model

The role of the individual, micro-, meso-, and macro - system in sport persistence			
**Individual level**	**Micro level**	**Meso level**	**Macro level**
**Non-psychological factors** • gender • level of education • age • socio-cultural and ethnic background • sport-related demographical variables (sporting habits, sport biography, year of experience / career length, placing and level of competition, exercise participation level, number of sporting hours, training patters **Psychological factors** • personality /perception of ability, self-efficacy, physical self-perception profile, perception of success, risk perception and perceived behavioural control, personal optimism, adaptive coping, competitiveness and perfectionism • motivation (extrinsic and intrinsic motivation, identified and introjected motivation, amotivation, self-determination theory) • orientation (goal orientation, social orientation, and win-orientation) • learning and development (learning strategies, development/progress, positive feedback, pursuit of learning and comparison) • commitment (personal investment, social constraints, involvement opportunities, valuable opportunities and other priorities) • positive feelings (enjoyment, satisfaction with sport practice, life satisfaction and openness) • negative feelings (lack of time, tiredness, exhaustion, and energy deficit) • future benefits (action planning and future sport intentions) • health (physical health and physical complaints, mental health, social, emotional and school-related functioning, fear of injury and having injury and worry about health	**General factors** • social acceptance • social support **Coach-related factors** • leadership style • motivational climate • coach’s interpersonal behaviours **Peer-related factors** • teammates to facilitate personal investment • task-oriented and ego-oriented perception of peers **Family-related factors** • parents’ investment • parental support • sibling influence **Neighbourhood** • neighbourhood remoteness • availability of parks and playgrounds	**Climate-related factors** • quality of coaching and support provided by sporting organisations • ego- and task-oriented climate • coach’s mastery and competitive climate • inclusive team environments, and opportunities for growth	**Cultural factors** • subjective norms • conflict with cultural expectations • beliefs in religious rules • the lack of opportunity or resources (e.g. lack of programs or facilities) • gender stereotypes (generally and toward sport participation)

The results concerning socio-cultural and ethnic background were less clear. In the studies, the social background of the respondents or their participating children was not clearly defined, only Consoni et al. [32] stated that they investigated adolescents belonging mostly to the middle class, however, the impact of the social status was not measured. Concerning sport persistence, there is a lack of investigations on the importance of social status, therefore, we can only consider the knowledge concerning its impact on sport motivation and sporting habits. The situation is the same when analysing the issue of ethnicity. Even investigations carried out in the USA did not state the ethnicity of the respondents. The nationalities were rather introduced following the country of origin of the papers. As an exception, Consoni et al. [32] stated that persistence was influenced by nationality (Italian students were more affected by dropout compared to Spanish athletes).

Only one study [38] declared that they investigated Latin and white non-Latin athletes. In the other cases, we received information only regarding their nationality. Most frequently, French [39; 40; 41], Australia [42; 43, 44], Canadian [50; 45; 46] were investigated in three-three studies. German athletes were represented in two papers [47; 48] were while one-one study explored the sport persistence of Estonian [49], Slovenian [51], and Italian [32] athletes. In four cases, we did not meet the nationality of the participants [35–37, 53].

Concerning sport-related demographical variables, we could detect the overrepresentation of factors reflecting on *elite sport*. In our interpretation, following the line of the papers analysed, the difference between elite athletes and non-elite athletes lies primarily in their level of performance, achievement, and dedication within their respective sports. Elite athletes typically excel at the highest competitive levels, often representing their country or professional teams, and are characterized by exceptional skills, physical prowess, and consistent success in competitions. They undergo rigorous training regimens, receive specialized coaching, and may derive substantial income from endorsements or competitions. In contrast, non-elite athletes participate in sports at recreational, amateur, or lower competitive levels, often for enjoyment, fitness, or personal fulfilment. While they may demonstrate proficiency and passion for their chosen activities, their involvement typically does not reach the pinnacle of achievement seen in elite athletes. Nevertheless, both groups share a common love for sports, dedication to improvement, and the pursuit of personal goals, albeit within different contexts and levels of competitiveness (Lorencz, 2013). Sport biography [47], year of experience [51] or career length [51] also refers to carrier-related factors. Also related to competitive sport, placing [51] and level of competition [47] are named as factors having significant impact on sport achievement and persistence. Exercise participation level [47] as well as number of sporting hours [47, 51] and training patters [46] can be categorized as individual non-psychological variables reflecting on type of behavioural commitment. Concerning elite athletic context, the relevance of language knowledge is also stated [43]. Unfortunately, we could detect only one paper mentioning the social disadvantage (Index of Relative Socio-economic Advantage and Disadvantage, IRSAD) as potential hindering factor concerning sport persistence [43]. Directly, the paper of Eime et al. [43] emphasises the relevance of sport facilities and their positive impact. Finally, we could detect participation in leisure-time sport activities as a potential factor influencing sport persistence mentioned in the study of Vella et al. [42] and Miller & Siegel [37].

#### Psychological factors

After the categorisation of the individual non-psychological factors, we continued the detection of the psychological variables. As a result of the investigation, we detected nine groups of variables namely personality, motivation, orientation, learning and development, commitment, positive feelings, negative feelings, future benefits and health.

*Personality* is significant concerning sport-related behaviour and sport achievement as well. Therefore, it is not surprising that most research grabbed sport persistence as an outcome of a personality-related variable. Mostly, perception of ability [33, 39, 43, 47], self-efficacy (including action, maintenance and recovery self-efficacy) [37] and physical self-perception profile [41, 43] were emphasised. Perception of success was also emphasised as a factor having a significant positive impact on commitment and persistence [33, 41, 48] which is also strongly dependent from confidence [37, 48]. Related to perception, risk perception [37] and perceived behavioural control [44] were also mentioned as supporting sport achievement and commitment. The relevance of the temperament was also measured in one study [42]. Positive psychological variables like personal optimism [48] and adaptive coping [37] also appeared as supporting factors. Although competitiveness [48] and perfectionism [48] can be regarded as double-edges swords, they were also highlighted. Concerning the self, the positive impact of the mindset [38], grit [38] and resilience [48] was also visualised.

As the second biggest group, *motivation* was detected, including extrinsic and intrinsic motivation, intrinsic motivation toward accomplishments and motivation to experience stimulation, identified and introjected motivation, amotivation] [36, 40, 45, 49, 50]. Related to motivation, the Self-determination theory appeared in more studies [ relatedness: 34,39, 40,49, competence: 33,34,36,37,39,40,41,49; autonomy: 34,39,40,49]. As specific outcome, body-related motivation [36] was explored as a factor having a significant impact on persistence. As the hall of motivation, behavioural intentions [37, 44] and basic psychological need [44] were investigated.

Besides motivation, various types of *orientation* appeared in the papers. As supposed, mostly achievement goal orientation [athlete’s task-orientation and ego-orientation] [38, 47, 53] could have been detected as the most important way of orientation. Besides, social orientation [47], and win-orientation [47] seemed to be significant and positive on persistence.

*Learning and development* is also relevant concerning long-lasting sporting activity. This included learning strategies [cognitive-metacognitive, affective motivational] [32], development/progress [39], positive feedback [35], the athlete’s pursuit of learning [38, 40, 41], and the athlete’s pursuit of Comparison [38, 40, 41, 53], all having a supporting impact.

*Commitment*, as often used as a synonym of persistence, of course, appeared as an important group of variables [35, 39, 49]. It included personal investment [34, 39], social constraints [39], involvement opportunities [39], valuable opportunities [39] and other priorities [39].

Concerning the detectable feelings, we were able to categorise both positive and negative ones. Regarding the *positive feelings*, enjoyment [36, 39], satisfaction with sport practice [35], life satisfaction [47] and openness [47] could have been coded, and all article reported the positive impact of these variables. On the contrary, the effect of the *negative feelings* including lack of time [43], tiredness [43], exhaustion [47], and energy deficit [47] negative impacts could have been recorded.

In some cases, the role of the *future* and plans could have been seen. The variables related to these groups were outcome expectancies [37], action planning [37] and future sport intentions [34, 40], highlighting the positive impact of planning and positive future-related thoughts.

Last but not at least, the relevance of *health* was detectable. This covered all aspects of health following the bio-psycho-social health model. Therefore, physical health [42] and physical complaints [47], mental health [42], various types of functioning [including social, emotional and school-related functioning, 42], fear of injury [43] and having injury [43] and worry about health [47] could have been seen as variables influencing persistence. As a result, we could see that better health [regarding all aspects] leads to higher level of persistence.

### The role of the micro-system in sport persistence

Concerning the micro-system level factors connecting to sport efficacy and persistence, general and specific factors could have been detected. Without specific categorisation, as general variables, *social acceptance* [35, 36] and *social support* [35] appeared, both factor having a positive impact and enhancing the level of sport persistence. Concerning specific groups, coach, family, peer and neighbourhood-related factors could have been categorised.

It is not surprising that coach-related factors appear to be significantly important. Higher levels of competitiveness as well as higher level of training and instruction [35] positively correlates to persistency. Obviously, leadership style is found to be an important factor. The results of Calvo & Topa [35] and Pelletier et al. [45] strengthen the fact that autonomy-supportive leadership style (regardless of coming from teachers, parents, coaches, school administrators or health care professionals) facilitates self-determined regulation, supporting persistency whereas a controlling style undermines self-determination. Sarrazin et al. [40] stated that task-involving motivational climate led to a higher perception of competence, sense of autonomy and relatedness, whereas the perception of an ego-involving motivational climate was not associated with positive impacts which was also visible in case of the papers measuring the task-oriented and ego-oriented perception of coach [33, 38, 40]. This also reflects on the positive impact of the coach’s interpersonal behaviours which helps to maintain persistence in case of open and supportive behaviour [45, 46]. Last but not at least, the personal investment of the athlete was already highlighted, however, coach’s investment is of paramount importance as well, having a positive impact on commitment [39].

Since adolescents and young adults were put in the focus of the research, the role of peers and teammates should be also emphasised. Usually, sport peers and peer influence have a strong supporting impact on motivation, commitment and persistence [46]. As visible in case of coaches as well, the perceived investment of teammates is also significant [39] since it can facilitate the personal investment of the athlete. Related to this, the task-oriented and ego-oriented perception of peers also appeared in the papers [33, 38, 40] which is related to the climate as well.

As a third big group, *family-related factors* could have been categorised which is also unsurprising knowing the fact that family is the primary socialisation area in childhood and remains still strong in adolescence and young adulthood as well. Therefore, parents’ investment [39] and parental support [46] have a significant positive impact on persistence. Besides, the role of the siblings can be also huge as sibling influence can also support commitment [46].

Finally, the relevance of *neighbourhood* was explored in some cases. Concerning this group, neighbourhood remoteness [43] and the availability of parks and playgrounds (PP) [42] were measured. The first reflects on the close environment integrating sport-related elements, neighbour and partly sport-related facilities while the latter is rather a factor referring to the infrastructure of the close environment. Both can increase the level of sport persistence on a long-term basis.

### The manifestation of the meso-system

As meso-level factor, *climate* could have been detected. Climate is considered a meso-level variable while it often reflects not only the close environment of the athlete (known as micro-level variables) but on the relationship of these environmental factors. In this regard, papers mentioned ego-oriented climate [33, 35, 38, 39, 41, 49] and task-oriented climate [33, 35, 38, 39, 41, 49] from the perspective of the athlete. The former emphasises social comparison and focuses on demonstrating superiority over others while the latter prioritises individual and collective improvement, learning, and mastery of skills. An optimal level of both is proved to be supportive in enhancing the level of sport persistence. From the coach’s side, coach’s mastery and competitive climate [34, 39] could have been detected, stating that coach-created mastery climate predicted psychological need satisfaction, self-determined motivation and commitment to sport.

### The relevance of the macro-system level factors behind sport persistence

It is not surprising that besides the meso-level factors, macro-level (basically cultural) factors influencing sport persistence are also underrepresented. Subjective norms were measured from the aspect of organised sport, indicating that satisfaction of basic psychological needs correlates with more positive attitudes, higher levels of perceived behavioural control, and more favourable subjective norms, which also predicts sport continuation [44]. Conflict with cultural expectations and beliefs in religious rules can be, however, hindering factors in sport commitment and persistence [42]. So are the lack of opportunity or resources, e.g. lack of programs or facilities, gender stereotypes (generally and toward sport participation) and gender stereotypes [rules about boys and girls playing together].

## Conclusions

Based on the ecological model, individual non-psychological and individual psychological, micro-level, meso-level and macro-level factors were categorised and introduced (Table 2). Overall, it is not very typical to emphasise the socio-demographic and career-related background variables as factors enhancing sport persistence which is surprising since sports participation during childhood can best be predicted by a combination of socioeconomic, cultural, and parental support variables. Therefore, involving sociodemograpic variables in such measurements would be important. In some cases, it turned out that *gender* can have a significant impact on sport persistence [42, 47], typically showing the higher persistence of male athletes which also reflects on previous research results concerning long-term sport participation [e.g., 43]. Also, measuring *socioeconomic status* was underrepresented. More specifically, only one paper had clear description about the social status and class where the research sample belong. Previous research show that children and youth coming from lower social status and worse financial situation as well as athletes having parents with lower educational level have significantly higher chance for dropping out of sport [54]. However, we could not detect any paper that explored the role of parental education, and they even did not mention this variable in their investigations. Also, age can be also crucial in sport persistence which is also dependent from the level of sport. Introducing the age of the participants is typical in studies and we could state the lack of this information only in some cases where rather age category was given by the authors. However, connecting to this line, the lack of the athlete’s educational level or status could be seen. It would be important to know the level of education in all cases since in some scenario, the education and sport participation of athlete students is supported parallelly following the dual career model [29].

Measuring the impact of the career-related variables would be also important at least in case of elite sport participation and persistence. Sport biography and experience is extremely important in case of elite athletes. Probably, due to their specific natures, no comparison was carried out between team sport and individual sports. Being a member of a team can have an additional positive impact on sport participation, but it also may depend on the level of sport participation [49]. Participation in leisure-time sport activities as a potential factor influencing sport persistence was measured [37, 42, 55]. However, it would be also paramount to explore competing activities and their role concerning sport persistence. Being employed, the type of work and the number of working hours as well as other constraints (e.g. voluntary work) may significantly influence sport participation and persistence, hypothetically in a negative way.

In our categorisation, the group of individual psychological variables was, unsurprisingly, the widest. We detected nine group of variables named as personality, motivation, orientation, learning and development, commitment, positive feelings, negative feelings, future benefits and health. These groups and variables belonging to them are the most typical factors investigated in papers focusing on sport participation, dropout and persistence. Personality-related variables, e.g., ability, self- perception, character and temperament, success, as well as coping, resilience or optimism are often in the crosshairs of research. Persistence is a positive variable – the higher it is, the more beneficial it is (of course, in an optimal interval). Therefore, it positively correlates with other positive variables (e.g., coping flexibility, optimism, self-assessment, self-image) and negatively with negative variables (e.g., anxiety, stress, pessimism, narcissism). It is also true when we investigate not only the correlation but the direction as well – positive variables have a positive, supporting impact which negative ones have a negative, hindering impact on sport persistence.

Motivation is an often-investigated psychological construct in psychology and pedagogy as well. Sport motivation plays a crucial role in sport persistence, which refers to an individual’s ability to continue participating in a sport over time [56]. It is the driving force that helps athletes maintain their commitment, overcome challenges, and stay engaged in their sporting endeavours. In the papers, its key components and correlates were also mentioned, e.g., intrinsic and extrinsic motivation, or the Self-Determination Theory (SDT) posits that three psychological needs—autonomy, competence, and relatedness—play a significant role in fostering intrinsic motivation and persistence. Commitment is closely linked to sport persistence and refers to an athlete’s dedication, loyalty, and perseverance in pursuing their sporting goals and continuing their involvement in their chosen sport. Commitment plays a crucial role in maintaining long-term engagement and overcoming obstacles.

In a strong relationship with motivation, a group of positive and negative feelings were detected. Overall, emotions play a significant role in sport persistence as they can influence an athlete’s motivation, performance, and overall engagement in their sport. Experiencing positive emotions, such as joy, happiness, and excitement, can enhance sport persistence. When athletes find pleasure and enjoyment in their sport, they are more likely to continue participating and remain committed over time [57]. These groups are only one step away from health which was our last group of variables detected. Physical, mental, and social health all play essential roles in sport persistence. Physical health is a fundamental pillar since when athletes are physically fit, they are better equipped to perform at their best, recover effectively, and avoid injuries. Physical health also affects an athlete’s energy levels and overall stamina, allowing them to endure demanding training sessions, competitions, and the rigors of their sport [58]. Mental health also plays a vital role in sport persistence as it influences an athlete’s emotional well-being, cognitive functioning, and overall mindset. Good mental health supports positive motivation and resilience, helping athletes stay committed and persist through challenges. It positively influences an athlete’s ability to focus, concentrate, and maintain mental clarity during training and competition. We should also emphasise that sport participation, especially at competitive level, can generate stress and pressure which also draws attention to the necessity of adaptive coping behaviour, mental toughness and health. Athletes with good mental health have the tools and strategies to manage stress effectively, maintain emotional balance, and prevent burnout. By managing stress levels, athletes can sustain their commitment and persist in their sport without experiencing excessive psychological strain [59]. Lastly, social health, which refers to the quality of an athlete’s relationships and social connections within their sporting community, is also fundamental. It is a variable on the border of the individual and micro-system levels. Having a supportive network of coaches, teammates, friends, and family helps the athlete to persist in their sport. Positive team dynamics, such as a supportive and cohesive team culture, can enhance sport persistence [60]. One may also not forget that sport is often a social activity, and positive social interactions can enhance an athlete’s enjoyment and overall experience.

Concerning the micro-system, following Bronfenbrenner’s categorisation, the relevance of the family, peers and coaches should be emphasised. The family is the primary socialisation area which is getting weaker in adolescence and youth but still represents essential values later in the life. The family can still provide emotional and practical support, which is particularly important in competitive sport and in childhood. At the same time, the role models and values shape an athlete’s values and attitudes towards sport. Positive family values, such as discipline, dedication, and perseverance, can provide a strong work ethic and foster long-term commitment to sport. Previous research have stated that the likelihood of sport participation is increased when at least one parent pursued sport previously [54]. Turning to the peers, they can be supportive in several ways. Peer relationships can offer encouragement, camaraderie, and a shared sense of purpose, which contribute to an athlete’s motivation and commitment. Interacting with peers can inspire healthy competition and push athletes to persist [55]. Peers provide opportunities for learning and growth through shared experiences, feedback, and collaboration. Last, but not at least, coaches play a vital role. They are essential in skill development and providing constructive feedback. When coaches provide effective guidance, technical expertise, and feedback, athletes can improve their performance, enhancing their motivation and persistence. Coaches also provide support and encouragement, serving as mentors and role models for athlete, and can play a crucial role in providing psychological support to athletes. They can help athletes manage stress, deal with setbacks, and develop mental resilience, all of which contribute to sport persistence [61, 62].

As meso-level factor, climate could have been detected which is, however, not a typical variable belonging to the meso-system. Meso-system factors refer to the interconnections and interactions between different settings and contexts that influence an individual’s development and behaviour [63]. However, due to its nature and content, we decided to put it into this group. As part of the climate, the quality of coaching and support provided by sporting organisations can influence sport persistence. Factors such as coaching styles, communication, and the organization’s commitment to athlete development play a role in shaping an athlete’s experience. Positive coaching practices, inclusive team environments, and opportunities for growth and advancement can enhance motivation and promote long-term commitment. Educational systems, social networks, and community resources can positively influence sport persistence. When an athlete has access to educational support systems, mentorship programs, and resources within their community, it can positively impact their overall well-being and motivation to persist [64, 65].

Lastly, macro-system factors, which refer to the broader cultural, societal, and institutional influences that shape individuals and their behaviours, were explored. Overall, subjective norms, cultural expectations and beliefs appeared in a few cases. However, these are also underrepresented in research focusing on sport persistence, even if the broader sport culture and community in which an athlete is embedded can shape sport participation [66]. A positive sport culture that values effort, sportsmanship, and personal development can foster a long-term commitment to the sport. On the other hand, a negative or toxic sport culture can discourage athletes and lead to disengagement.

Regarding the content of papers focusing on sport persistence, we could therefore find some topic missing or underrepresented. At the level of the individuum, variables reflecting on the *sporting career and experience* should be considered in all cases. *Sport biography* was mentioned only in one paper which is a huge hiatus since biography represents all the positive and negative events in the athlete’s life including scholarships, awards, and even injuries. All can have a paramount impact on persistent behaviour [67]. The literature also states that working with professionals e.g., sport psychologist can significantly enhance sport persistence [68]. Nevertheless, none of the papers involved in the systematic research focused on this topic which can mean that involving a sport psychologist is still not rooted in the sport practice or the impact of the supporting professionals was not considered in the studies. Regarding the micro- and meso-system, sports club membership was regularly investigated (it was more relevant compared to the level of education), however, the *relationship between the sports club/association and the school* was not investigated although it is well-known that a strong relationship between the school and the sport association (usually following the dual career model) can also contribute to the sport continuation of athletes, especially in case of competitive sport [26]. It was also surprising that no university sports clubs could have been detected in the studies, which suggests that sport persistence, above a certain age, is rather independent of schooling, although we have to emphasise that elite athletes can also study in higher education, university clubs can also participate in elite competitions and sport participation as leisure activity should be important in all life stages (including higher education) as well. The *classic meso-system level factors* like family-school relationship, family-coach relationship is missing from such research. Previous investigations showed that the integration of school and sport can have a direct impact on sport persistence [69]. When there is a positive relationship between an athlete’s academic pursuits and their sport, it can foster a sense of balance and synergy. Supportive policies, flexible schedules, and understanding from school authorities can enable athletes to manage their academic responsibilities alongside their sport, promoting persistence. The support and involvement of parents or guardians are influential in sport persistence. Positive parental involvement, including emotional support, encouragement, and logistical assistance, can have a significant impact on an athlete’s motivation and commitment. Parental support also extends to ensuring a healthy balance between sport and other aspects of life, facilitating overall well-being and persistence. These results confirm that meso-system should be also represented in sport persistence research as well.

Concerning methodological issues, we have to emphasise that except for one interview study, only cross-sectional studies could have been detected. No randomized controlled trials (RCTs), non-randomized controlled trials (NRCTs) or cluster randomized trials (CRTs) were found in the topic which implies that none of the previous research focused on the introduction and evaluation of programs developed for enhancing and maintaining sport persistence. However, this would be significantly important from several aspects. Sports persistence programs provide individuals with the opportunity to develop and improve their athletic skills. Concerning the psychological skill-related aspect, persistence programs can encourage consistent effort, athletes can learn how to set both short-term and long-term objectives. They can help shape individuals’ character by fostering important qualities such as discipline, perseverance, teamwork, and resilience, leading to long-term engagement. Last, but not at least, sports persistence programs often foster a sense of camaraderie and community among participants [68].

Although this review offered a detailed comparison between the studies included in the literature, the research is not without limitations. Only studies that investigated sport persistence were included, which may not lead to a comprehensive understanding of its development. Only cross-sectional studies could have been detected which did not allow us to investigate its changing nature. Also, the lack of methodological diversity hindered us in detecting specific programs focusing on the development of sport persistence. Only English papers were included, which can also be a hindering factor in detecting such programs. While we did gather extensive data on factors such as the quality of the trial, the participants, and the interventions, there remained some unaccounted-for heterogeneity in the trials. Due to the heterogeneity of the studies, no pool sizes and effect sizes could be measured.

Future studies should also focus on the topics detected as underrepresented themes to better understand the nature of sport persistence and our potentials to its development and maintenance. Another essential aspect would be the clear definition of the level of sport in each paper. Most articles only mention the category of athletes (i.e. elite- and/or non-elite athletes) without the further description of the nature of their sporting activity. In some cases, it can be problematic, especially at younger age, until approximately 12 years, in some sports and countries the competitions organised represent only at least the regional level, and national competitions at that age are not organised [70]. Furthermore, longitudinal investigations should be also carried out to reach a better understanding.

## Electronic supplementary material

Below is the link to the electronic supplementary material.

Supplementary Material 1

## Data Availability

Data are available only on request due to ethical restrictions.

## Abbreviations

CRT: or cluster randomized trial
EBSCO: Elton B. Stephens Company
NRCT: Non-randomized controlled trials
PRISMA: Preferred Reporting Items for Systematic Reviews and Meta-Analyses
RCT: Non-randomized controlled trial
WHO: World Health Organisation
