# Supplementary material for: Factors influencing sport persistence still represent a knowledge gap – the experience of a systematic review
Source: BMC Psychol. 2024 Oct 23;12:584. doi: 10.1186/s40359-024-02098-6 (PMC11515643; doi:10.1186/s40359-024-02098-6)
Supplement: Supplementary file 1 — Supplementary Material 1 [file 40359_2024_2098_MOESM1_ESM.docx]

**Appendix**

Table 1. The evaluation of the risk of bias of the papers based on Joanna Briggs Institute (JBI) critical appraisal tool (Moola et al., 2020)

|  | **Q1** | **Q2** | **Q3** | **Q4** | **Q5** | **Q6** | **Q7** | **Q8** | **Risk assessment** |
| --- | --- | --- | --- | --- | --- | --- | --- | --- | --- |
| Ahn et al (2016) | No | No | Yes | Yes | Yes | Yes | Yes | Yes | Low |
| Albert et al. (2019) | Yes | Yes | Yes | Yes | Yes | Yes | Yes | Yes | Low |
| Baron-Thiene & Alferman (2015) | Yes | Yes | Yes | Yes | Yes | Yes | Yes | Yes | Low |
| Bars et al. (2009) | Yes | Yes | Yes | Yes | Yes | Yes | Yes | Yes | Low |
| Calvo & Topa (2019) | Yes | Yes | Yes | Yes | Yes | No | Yes | Yes | Moderate |
| Cervelló et al (2007) | Yes | Yes | Yes | Yes | Yes | Yes | Yes | Yes | Low |
| Consoni et al. (2021) | Yes | Yes | Yes | Yes | Yes | Yes | Yes | Yes | Low |
| Duda (1988) | Yes | Yes | Yes | Yes | No | No | Yes | Yes | Moderate |
| Eime et al (2014) | Yes | Yes | Yes | Yes | Yes | Yes | Yes | Yes | Low |
| Fraser-Thomas et al. (2008) | Yes | Yes | Yes | Yes | No | No | Yes | Yes | Moderate |
| Gucciardi & Jackson (2015) | Yes | Yes | Yes | Yes | Yes | Yes | Yes | Yes | Low |
| Guillet et al. (2002) | Yes | Yes | Yes | Yes | Yes | Yes | Yes | Yes | Low |
| Joessar et al. (2011) | Yes | Yes | Yes | Yes | Yes | Yes | Yes | Yes | Low |
| Lea & Branco (2020) | No | No | Yes | Yes | Yes | Yes | Yes | Yes | Low |
| Lukwu & Luján (2011) | Yes | Yes | Yes | Yes | Yes | Yes | Yes | Yes | Low |
| Miller & Siegel (2017) | Yes | Yes | Yes | Yes | Yes | Yes | Yes | Yes | Low |
| Pelletier et al. (2002) | Yes | Yes | Yes | Yes | Yes | Yes | Yes | Yes | Low |
| Ryan et al. (1997) | No | No | Yes | Yes | No | No | Yes | Yes | Moderate |
| Sarrazin et al (2002) | Yes | Yes | Yes | Yes | Yes | Yes | Yes | Yes | Low |
| Vella et al. (2014) | Yes | Yes | Yes | Yes | Yes | Yes | Yes | Yes | Low |
| Westmattelmann et al (2021) | Yes | Yes | Yes | Yes | Yes | Yes | Yes | Yes | Low |

Note: Questions of JBI are as follows: Q1. Were the criteria for inclusion in the sample clearly defined? Q2. Were the study subjects and the setting described in detail? Q3. Was the exposure measured in a valid and reliable way? Q4. Were objective, standard criteria used for measurement of the condition? Q5. Were confounding factors identified? Q6. Were strategies to deal with confounding factors stated? Q7. Were the outcomes measured in a valid and reliable way? Q8. Was appropriate statistical analysis used?
